# Potential surgical therapies for drug‐resistant focal epilepsy

**DOI:** 10.1111/cns.13690

**Published:** 2021-06-07

**Authors:** Wei Shan, Xuewei Mao, Xiu Wang, Robert E. Hogan, Qun Wang

**Affiliations:** ^1^ Department of Neurology Beijing Tiantan Hospital Capital Medical University Beijing China; ^2^ National Center for Clinical Medicine of Neurological Diseases Beijing China; ^3^ Beijing Institute for Brain Disorders Beijing China; ^4^ Beijing Key Laboratory of Neuro‐modulation Beijing China; ^5^ Shandong Key Laboratory of Industrial Control Technology School of Automation Qingdao University Qingdao China; ^6^ Departments of Neurology and Neurosurgery School of Medicine Washington University in St. Louis St. Louis MO USA

**Keywords:** epileptogenic, laser‐induced thermal therapy, magnetic resonance‐guided, neuromodulation

## Abstract

Drug‐resistant focal epilepsy (DRFE), defined by failure of two antiepileptic drugs, affects 30% of epileptic patients. Epilepsy surgeries are alternative options for this population. Preoperative evaluation is critical to include potential candidates, and to choose the most appropriate procedure to maximize efficacy and simultaneously minimize side effects. Traditional procedures involve open skull surgeries and epileptic focus resection. Alternatively, neuromodulation surgeries use peripheral nerve or deep brain stimulation to reduce the activities of epileptogenic focus. With the advanced improvement of laser‐induced thermal therapy (LITT) technique and its utilization in neurosurgery, magnetic resonance‐guided LITT (MRgLITT) emerges as a minimal invasive approach for drug‐resistant focal epilepsy. In the present review, we first introduce drug‐resistant focal epilepsy and summarize the indications, pros and cons of traditional surgical procedures and neuromodulation procedures. And then, focusing on MRgLITT, we thoroughly discuss its history, its technical details, its safety issues, and current evidence on its clinical applications. A case report on MRgLITT is also included to illustrate the preoperational evaluation. We believe that MRgLITT is a promising approach in selected patients with drug‐resistant focal epilepsy, although large prospective studies are required to evaluate its efficacy and side effects, as well as to implement a standardized protocol for its application.

## Graphical abstract

The figure exhibits diagnostic and therapeutic treatment approach to MRgLITT with drug‐resistant epilepsy (DRE). The inclusion of stereotactic EEG (SEEG), MRI‐guided laser interstitial therapy (MRgLITT) as they pertain to the diagnostic and therapeutic management with DRE. ECoG, electrocorticography; SDG, subdural grid.

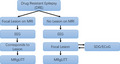

## 1 INTRODUCTION

Focal epilepsy is caused by the abnormal electrical discharges (identified by intracranial electrophysiological recording) in specific focus of the brain (originated in only one part of the brain, namely epileptogenic zone).^1^ Drug‐resistant focal epilepsy is diagnosed after two proper anti‐epilepsy drugs have failed.^2^ The presence of drug resistance is typically unpredictable, although some believe that peripheral DNA methylation signatures and microRNA may help.^3^, ^4^ Although the pathogenesis of drug‐resistant focal epilepsy remains unclear, several studies proposed that genetic predisposition plays an important role.^5^, ^6^, ^7^, ^8^

Patients with drug‐resistant focal epilepsy are more suitable for surgical operation, and are more likely to benefit from removal of the culprit tissue.^9^, ^10^, ^11^ An extensive preoperative evaluation should then be conducted, including clinical symptoms, underlying brain conditions (such as brain infection, chronic syndrome, neurofibromatosis, tuberous sclerosis, brain tumor, stroke, and blood vessel malformations), medical history, blood tests, cerebrospinal fluid (CSF) analysis, neuropsychology testing, electroencephalography, and imaging scans. Available neuroimaging scans include CT scan, MRI scan, positron emission tomography, single photon emissions computerized tomography (SPECT), and magnetoencephalography (MEG).^12^, ^13^, ^14^, ^15^, ^16^, ^17^, ^18^, ^19^ Importantly, the preoperative evaluation should try to pinpoint the epileptogenic region which may adjoin or overlap with brain areas responsible for language, memory, movement, and emotion, in order to avoid or minimize the impact on the surrounding normal brain tissues during epileptic surgery. Should the concordance be established among intracranial electrophysiology, structural MRI and pathology, a suspected epileptogenic zone is identified accordingly, and the patient should be offered the choice to have the epileptogenic zone resected. Drug‐resistant focal epilepsy surgery should follow the “3M principle”: (1) “M”aximum removal of structural brain lesions (i.e., malformations of cortical development^20^ and low‐grade neoplasms^21^); (2) “M”inimum injury to neurologic function^13^, ^22^ ; and (3) “M”aximum recovery to control seizures without inducing other morbidities.^17^, ^19^, ^20^ These resections may not only involve of the medial structures of the temporal lobe such as the amygdala, hippocampus, and entorhinal cortex, but also involve the neocortex of the temporal and other lobes.^23^ Resections of the cortex are guided by imaging results and intracranial electroencephalography.^24^, ^25^, ^26^, ^27^

With decades of development and safety control applications, laser‐related surgery in neurosurgical patients has become significantly safer.^28^, ^29^, ^30^, ^31^ In recent years, a combination of integrated laser‐induced thermal therapy (LITT) with magnetic resonance imaging (MRI), termed magnetic resonance‐guided laser‐induced thermal therapy (MRgLITT), has been introduced to support image‐guided surgery (IGS) and intraoperative imaging (IOI).^32^, ^33^, ^34^, ^35^, ^36^, ^37^ MRgLITT serves as a novel option for lesionectomy of the seizure‐onset zone, and in addition, facilitates advanced disconnection procedures for intractable epilepsy.^33^, ^35^, ^38^, ^39^, ^40^

Thus, in the present review, we will first briefly introduce drug‐resistant focal epilepsy and its clinical evaluation approaches. We will then introduce the potential alternative therapies for drug‐resistant focal epilepsy, such as epileptogenic foci resection, vagus nerve stimulation, reactive nerve stimulation modulation surgeries, and deep brain stimulation modulation surgery. And finally, focusing on MRgLITT, we will extensively discuss its technical issues, clinical usage, and safety control.

## 2 EPILEPSY AND ITS CLINICAL EVALUATION

### 2.1 Prevalence and incidence of epilepsy and drug‐resistant epilepsy (DRE)

Epilepsy is a common CNS disorder epidemiologically, with a prevalence of 6.38 per 1000 persons, and lifetime prevalence 7.60 per 1000 individuals.^41^ Its annual cumulative incidence is 67.77 per 100,000 individuals, and the incidence rate is 61.44 per 100,000 person‐years.^42^

Among the entire epileptic patients, approximately 20%–30% will gradually show drug resistance, defined by persistent seizures despite administration of two antiepileptics with an active and well‐tolerated dosage exactly adapted to the requirements of patients,^42^, ^43^ either sequentially or in combination. DRE has become one of the major public health issues. Among such patients, those with an identifiable epileptogenic lesion (drug resistance focal epilepsy) are good candidates for surgical remediation, traditionally with open resection. However, there are still considerable limitations for traditional surgical approaches, such as high requirement of overall health status, easy access to the location of pathological tissue and high comorbitidies.^44^

### 2.2 Drug‐resistant focal epilepsy (DRFE)

Focal epilepsy (formerly known as partial seizures) refers to the electrical and clinical manifestations of seizures that arise from one portion of the brain.^45^, ^46^ An electroencephalogram typically reveals a localized discharge over the area of onset, or regions beyond the initial onset as the abnormal electrical activity propagates. Focal seizures can originate from any lobe in the brain, with temporal lobe as the most frequently recognized origin.^46^ Drug‐resistant focal seizure should be considered in those whose seizure remission is not achieved after two monotherapy trials followed by a dual therapy trial.^9^ Thorough evaluations should be performed to confirm the diagnosis and to consider of resective epilepsy surgery and/or neuromodulation therapies.^47^, ^48^

In adults, the presence of focal seizure strongly implies an underlying focal structural lesion (e.g., stroke, brain tumor).^49^, ^50^, ^51^, ^52^, ^53^, ^54^ In contrast in children with focal seizures, only 10 percent have brain tumors or strokes, and no focal structural lesion is present in the majority of patients, in with the seizure is either cryptogenic^55^, ^56^ or as the manifestation of an idiopathic disorder (benign rolandic epilepsy).^57^, ^58^, ^59^ The behavioral manifestations (seizure symptoms) of focal seizures relate not only to the region of the brain involved during the ictal discharge, but also to the maturity of the nervous system and the integrity of the pathways (neural circuit) necessary for clinical symptom's pattern.^60^, ^61^, ^62^ This is particularly true in infants and children with diffuse encephalopathies, in whom brain immaturity, diffuse cerebral dysfunction, or both make manifestations of focal seizures difficult to recognize.^62^ Focal seizures also can be mistaken in older children when the presence of secondary convulsive movements prompts casual observers to label the event a “generalized tonic‐clonic” seizure.^60^, ^61^

### 2.3 Clinical evaluation for epilepsy surgery

Epileptogenic focus resection and regulation/modulation are functional neurosurgeries with certain risks, such as intracranial bleeding, infection, allergic reaction to the anesthesia, function loss due to the brain tissue remove (vison loss, speechless, memory loss, or movement problem), and lesion tissue residue induced recurrence of seizure.^62^, ^63^, ^64^, ^65^, ^66^ Therefore, to make the final suggestion for the individualized resection, ablation, or modulation, the candidates must be strictly selected based on surgery indications, and the epileptic area should be accurately allocated the through comprehensive preoperative evaluations. The next step is to appropriately establish the surgical strategy, including the resection procedure and all regulatory modalities, in order to improve the surgical efficiency, reduce the surgical complications, and strengthen the comprehensive management postoperatively.^66^

**FIGURE 1:**
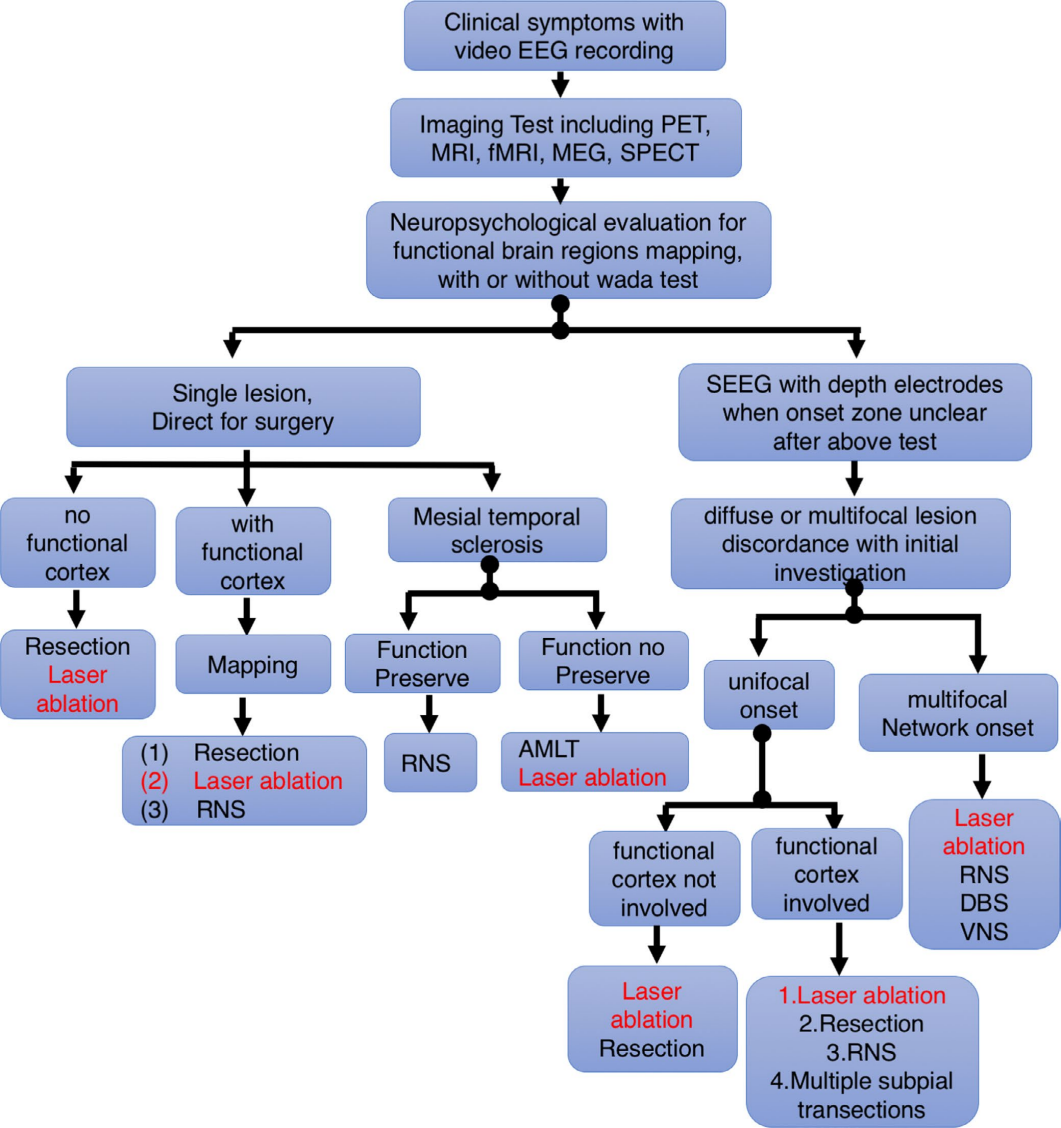
Surgical decision tree used in Beijing Tiantan Hospital Epilepsy Center. The findings from non‐invasive investigations determine whether a patient is referred directly to surgery (single lesion) or to intracranial study (diffuse or multifocal lesion, normal MRI, or discordance). In patients referred directly to surgery, findings from non‐invasive investigations (involving functional cortex or mesial temporal sclerosis) determine surgical treatment

A detailed preoperative the clinical evaluation should include the following: (1) The medical history and previous epilepsy care and treatment should be reviewed by an epilepsy specialist.^67^, ^68^, ^69^ (2) Results of monitoring and imaging tests with episodes recorded with video and electroencephalogram (EEG) monitoring. If a single abnormal brain area is identified, it is likely the epileptogenic zone.^70^, ^71^, ^72^ (3) MRI examination which could provide abundant visible details to identify even subtle brain abnormalities that may relate to the seizure.^55^, ^73^, ^74^, ^75^ (4) PET provides the location and presence of brain metabolic disturbances, and might pinpoint the tissue responsible for seizures.^76^, ^77^, ^78^ (5) SPECT provides a remarkable "snapshot" of brain activity in brain locations through measuring the blood flow.^76^ (6) MEG has much greater resolution than typical with EEG. MEG results can be combined with MRI and other brain imaging to provide a very comprehensive view of the brain function and structure.^79^, ^80^ (7) Neuropsychology testing provides information on the patient's language, memory, and comprehension skills. Wada test may also be applied to evaluate the speech and memory functions and to set the dominant area for these crucial functions.^81^, ^82^ (8) Intracranial monitoring comes with a more confirmative idea on the epileptogenic zone, in comparison with all the abovementioned tests.^83^, ^84^ (9) Epilepsy surgery conference^85^, ^86^ held by a team of epileptologist, neurosurgeons, neuropsychologists, and nurses will go over all aspects of evaluation and provide the best treatment options for the patient (Figure 1).

## 3 POTENTIAL ALTERNATIVE THERAPIES FOR DRUG‐RESISTANT FOCAL EPILEPSY

### 3.1 Epileptogenic foci resection

Resection surgery is the most developed and mature epilepsy surgery.^87^, ^88^ Ideally, postoperative patient should achieve complete remission of clinical attacks. The premise of operation is to clearly locate the epileptogenic area and functional area, and the epileptogenic area is relatively limited and not in close proximity to any important functional areas. Available procedures include the following: (1) Resection of medial temporal lobe structure,^89^, ^90^, ^91^, ^92^ which is a classic operation for the treatment of medial temporal lobe epilepsy. It is suitable for the epileptogenic areas in one side of the temporal lobe, or with clear structural boundaries in the temporal cortex. The extent of surgical resection was about 5cm backward of the temporal pole in the dominant temporal lobe, and 6cm backward of the anterior temporal lobe in the non‐dominant temporal lobe. Generally, the extent of posterior resection does not exceed the ipsilateral Labbe's vein. (2) Selective amygdalohippocampal resection.^93^, ^94^ It is suitable for simple medial temporal lobe epilepsy. The surgical approach can be through the lateral ventricle, temporal pole, lateral fissure, or temporal floor. (3) Neocortical resection,^95^, ^96^ which is suitable for partial epilepsy caused by focal and noncongenital lesions, such as space occupying lesions and trauma. Through accurate localization of the epileptogenic area, the resection can achieve satisfactory outcome. It is better to remove the tumor under the pia mater to protect the subcortical white matter from damage. (4) Multiple lobectomies,^97^, ^98^ which are suitable for patients with obvious brain structural abnormalities involving multiple lobes, resulting in multiple epileptogenic areas. The extent of resection depends on the nature and degree of the lesion, the size of the epileptogenic area, and its boundary with the functional area. Generally speaking, as long as the functional area is not damaged, the more thorough the resection of the lesion, the less likely the recurrence of seizure after surgery. (5) Hemispherectomy,^99^, ^100^, ^101^ which is used in limited conditions, such as hemiconvulsion‐hemiplegia‐epilepsy (HHE), unilateral hemispheric brain perforation, unilateral diffuse cortical dysplasia, Sturge‐Weber syndrome, and Rasmussen's encephalitis. Hemispherectomy options include anatomical hemispherectomy (modified operation) and functional hemispherectomy.

### 3.2 Vagus nerve stimulation/reactive nerve stimulation modulation surgeries

Vagus nerve stimulation (VNS)^102^, ^103^, ^104^, ^105^, ^106^ and responsive nerve stimulation (RNS) belong to neuromodulation surgery.^107^, ^108^, ^109^ They have similar mechanism but are different in the trigger regions. VNS stimulates the vagus nerve, while RNS stimulates the reactive brain local regions.^102^, ^107^ First reported in 1990, VNS was approved in 1997 by the Food and Drug administration (FDA) for the treatment of intractable epilepsy.^110^, ^111^ By 2014, more than 100,000 patients worldwide had received VNS stimulation, with an effective rate of 70%. It is mainly used for children or adults with limited drug‐refractory epilepsy (such as Dravet syndrome in children, and post‐trauma epilepsy in adults) but are not good candidates for surgical resection.^103^, ^112^ As for the procedure, first, a coil is placed on the vagus nerve in the left neck, and the stimulation device is buried in the chest. Then, in each outpatient visit, the medical staff will adjust the parameters and modes of the stimulation device through an instrument. It is found that the stimulation of the vagus nerve can improve the mood, consciousness, and memory in some patients, and thus further improve the quality of life in epileptic patients. It has been internationally recognized as a safe and effective treatment for children and adults with local and comprehensive intractable epilepsy, and its adaptive population is continuously increasing.^102^, ^104^, ^110^, ^111^, ^113^, ^114^

RNS was approved by the U.S. Food and Drug Administration (FDA) in 2013.^115^ This system is similar to a heart pacemaker. It can monitor brain waves, then respond to abnormal activities especially those seizure‐inducing activities. It has shown to reduce seizures and improve quality of life in most patients.^115^, ^116^, ^117^, ^118^ Tiny wires or leads are placed in one or two areas on the brain surface where seizure activity may originate. These wires connect to the stimulator placed in the skull, where the system can release small pluses or bursts of stimulation to the brain when anything unusual is detected. These pluses or bursts can stop the epileptogenic activity even before the seizure begins, or before the focal seizure spreads into a generalized seizure.^115^

The advantages of the modulation operation include the following: (1) no need to accurately locate the epileptogenic focus,^117^, ^118^ (2) less trauma leading to less adverse effects,^117^, ^118^ (3) adjustable mode with long‐term effect,^108^, ^117^, ^118^ and (4) efficacy up to 70%, associated with improvement in mental, emotional, cognitive function, and life quality in most patients.^119^ Indications for the VNS and RNS treatment include the following: (1) Diagnosis of unresectable multifocal epilepsy.^114^ (2) Focal epilepsy involving defined functional areas in patients could be involved in these treatments.^114^, ^115^, ^117^ (3) Unclear epileptogenic location after thorough clinical pre‐operational evaluation.^114^, ^115^, ^117^ (4) Recurrent seizure after the operation.^114^, ^115^, ^117^ (5) Patients who are not willing to open the skull.^114^, ^115^, ^117^ And (6) Total and partial epilepsy of unknown causes.^114^, ^115^, ^117^ In the past, VNS/NRS surgery could only be performed in epilepsy patients of 12–60 years of age. But nowadays, the range has been expanded to children over 2 years old, especially for the Dravet syndrome patients.^119^ Detailed information about research progress of VNS, RNS, and ANT‐DBS (deep brain stimulation [DBS] at the anterior nucleus of the thalamus [ANT]) in epilepsy treatment, including meta‐analysis on clinical trials, indications, possible mechanisms, efficacy, safety issues, and adverse effects could be found from our reports and others.^114^, ^120^

### 3.3 Deep brain stimulation modulation surgery

In DBS modulation surgery, DBS device is surgically placed in the indicated location, where thin electrodes carry electrical pulses from a nerve stimulator powered by a battery.^120^, ^121^ It can be programmed like a microcomputer (similar as a pacemaker). Instead of allowing for the free transmission of the epileptic current, the DBS is programmed to transmit the artificial current in a preset cycle. In this way, some epileptic circuits could be blocked to prevent the seizure or to reduce the seizure frequency.^120^, ^122^, ^123^, ^124^ In 2018, the FDA approved DBS of the ANT, namely ANT‐DBS for the treatment of drug‐resistant focal epilepsy patients (DRFE) when surgery or minimally invasive neuromodulation therapy is not possible or fails. DBS is designed to cure certain forms of epilepsy (including drug resistance focal epilepsy).^123^, ^125^ To determine whether a patient can benefit from DBS, a thorough evaluation should be conducted. If surgical removal of the epileptogenic region is not doable, a device such as RNS, VNS, or DBS can be considered.^114^ The clinical evaluation of DBS in the treatment of epilepsy takes into consideration the type of seizure in the patient, the best way to limit the risk of surgery, and the best way to provide the maximum benefit of DBS.^123^, ^124^

At present, the most common method to place DBS electrodes into the ANT is through direct neurosurgery, where the mammillothalamic tract (MTT) is an important anatomical landmark.^126^, ^127^ MTT is a prominent white matter tract that originates from the mammillary body and ends in the midgut of ANT where it connects the inner and outer layers of the thalamus, also known as the ANT‐MTT junction.^127^ In the Papez circuit which controls the emotional expression, ANT mainly receives the afferent information from the hippocampal formation through MTT, which connects with the cerebral cortex through thalamic radiation and thalamic cingulate fibers. Information from usage of DBS in treating dyskinesia shows that patient selection and electrode placement are important factors for clinical outcomes, which is very likely to be true in the case of epilepsy.^128^ Therefore, suggested key points for seizure control by DBS should include patient characteristics, such as seizure location and stimulation site. Besides, data from the SANTE (Stimulation of the ANT in the Treatment of Epilepsy) trial showed that DBS electrodes do not always have to be placed in ANT; rather, effective stimulation could be achieved from external contact with the ANT.^129^, ^130^ Therefore, the best stimulus point is still under investigation. It is speculated that ANT‐DBS prevents the spread of epilepsy and/or regulate the epileptogenic focus through its connection with the Papez circuit, although its exact mechanism of action and to what extent different brain networks and fiber tracts are stimulated remain unclear.^114^, ^129^ The importance of Papez circuit as potentially epileptic need to be confirmed by deep recording in humans and animal models. Failure of ANT‐DBS for epileptic control may be related to failure of MTT stimulation.^128^, ^129^, ^130^

Results from the SANTE trial also showed that bilateral thalamic stimulation is a safe surgical procedure for refractory focal epilepsy.^130^ It reduces the frequency of both short‐term and long‐term seizures and significantly improves the well‐being of patients. This was later confirmed by several cohorts, with an average response rate around 50% by one‐year since the initiation of ANT‐DBS treatment. In addition, the degree of epileptic control varies greatly from individual to individual. Notably, the positive effects of DBS treatment may not be immediately apparent. Like other neuromodulator devices, DBS treatment needs time to give full play to its advantages.^114^, ^118^, ^123^ Over time, seizures in a good portion of patients could improve significantly. DBS is usually used in combination with anti‐epileptics. Similar as with other anti‐epileptic devices, if DBS can improve symptoms, epilepsy drugs may be tapered.^114^, ^123^

In summary, resection and modulation surgeries serve as promising alternative approaches for DRE. In the next section, we are going to thoroughly review another technique, LITT and MRgLITT, as a novel alternative approach for the management of DRE.

## 4 LASER‐INDUCED THERMAL THERAPY (LITT)

### 4.1 History on surgical use of laser and LITT technology

Lasers have been used in specialized neurosurgeries for more than 50 years. In 1966, neurosurgeons began to use ruby lasers to treat malignant gliomas,^131^ and in 1969, carbon dioxide (CO_2_) lasers were used during the treatment of recurrent glioblastoma multiforme (GBM).^132^ Although lasers played a constructive role in the field of neurosurgeries, its clinical usage was initially very limited due to the lack of quality control and a real‐time monitoring system. Moreover, the large size of laser delivery systems and the big bulk of lasers made it unsafe in treating tumors deeply located within tissues.^131^ In 1980, its extensive medical application began with development of the neodymium‐doped yttrium aluminum garnet, Nd:Y_3_Al_5_O_12_ (Nd:YAG).^133^, ^134^ Since the Nd:YAG laser can be delivered with a pliable fiber‐optic cable while deeply penetrate neural tissues, the smaller trauma makes it much easier to achieve coagulation and hemostasis.^135^

As a kind of nonionizing radiation, laser can produce collimated and coherent beams of light energy. Parameters such as scatter and absorption are usually applied to determine the effectiveness of a laser on tissues.^136^, ^137^, ^138^ Absorption usually occurs after laser photons hit the target tissue molecules, which produces heat and eventually forms chromophores.^139^ When the energy is transferred to chromophores, the released heat will induce direct photothermal damages to adjacent tissue.^140^ During the interaction between photons and the particles within the cells or tissues, the trajectories of photons can be deviated. Scatter will then occur and increase spatial distribution of light.^141^ Based on the properties of target tissue, to achieve the optimal selective photothermolysis, the wavelength of photon scatter should be carefully selected to match the absorption for better tissue heating and light penetration.^142^, ^143^ Besides, specific tissue properties that may affect ablation should also be taken into consideration, such as the perfusion, the conductivity, the tissue‐specific treatment temperature, and the density.^142^, ^143^, ^144^

**FIGURE 2:**
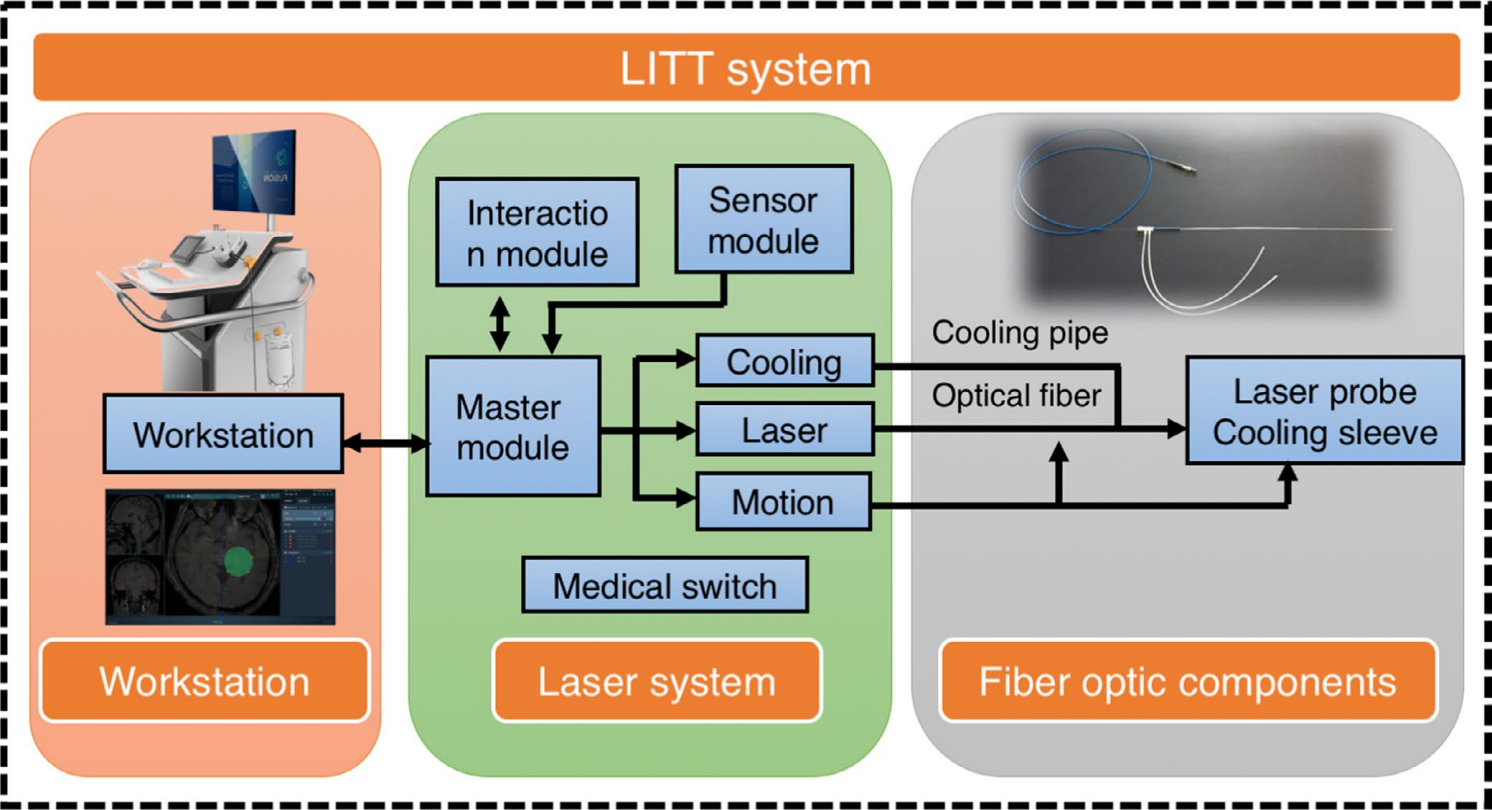
Structure of the MRgLITT units. The instruction included MR workstation, laser system, fiber optic components

**FIGURE 3:**
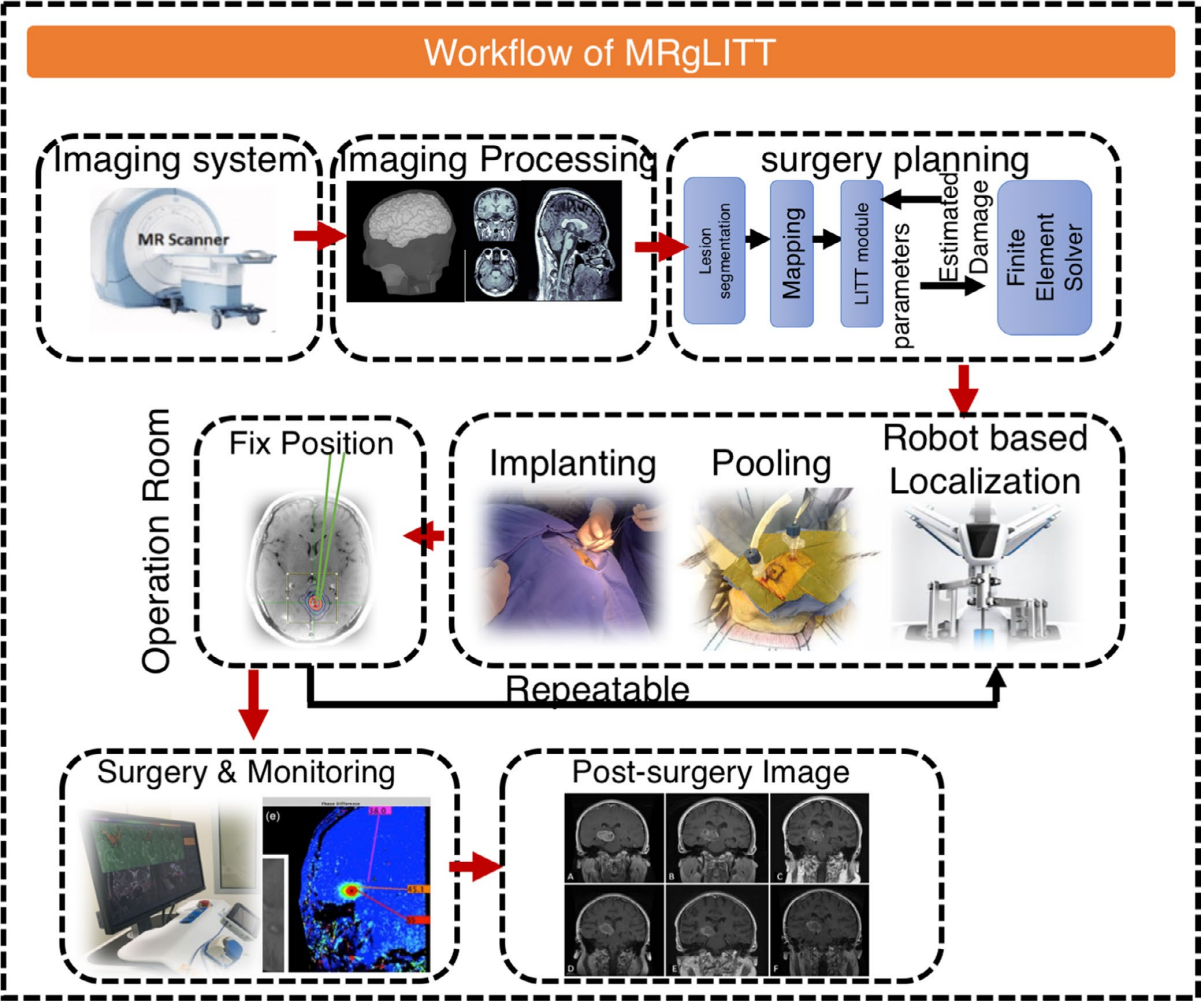
Operation workflow of the MRgLITT in the clinical practice. The workflow of MRgLITT before surgery involves imaging system, imaging processing, and surgery planning. In the operation room, the target area position is identified through implanting, pooling, and robot‐based localization, followed by surgery and simultaneous monitoring. A post‐surgery imaging is often required

LITT refers to the technique that delivers laser through optical fibers and irreversibly ablates the target tissue by heat. The fibers should be long enough to connect the patient with an outside laser source. During the LITT process, a diffusing tip with a length of approximately 1 cm is usually applied to introduce laser light into the patient's tissue.^145^, ^146^ To visualize the target tissue, novel imaging techniques such as magnetic resonance (MR) thermography can be combined with LITT. This allows surgeons to conduct laser trajectory planning to optimize laser position and implement real‐time assessment on the thermal damage (Figures 2 and 3).

### 4.2 Magnetic resonance‐guided laser‐induced thermal therapy (MRgLITT)

Lacking control of laser‐induced thermal damage on paraneoplastic tissues restricted the application of laser therapy for decades.^147^ The strait persisted until LITT was integrated with magnetic resonance imaging (MRI), which enables surgeons to accurately estimate and monitor the thermal damage, and to operate on lesions deeply located in brain.^148^, ^149^, ^150^ This innovative technique is called MRgLITT, also known as magnetic resonance‐guided stereotactic laser ablation (MRGSLA). As a minimally invasive procedure, it revolutionizes application of laser in assorted focal lesion treatments with real‐time intraoperative imaging monitoring.^151^, ^152^, ^153^

MRgLITT involves positioning the patient's head within a stereotactic frame and guiding a laser emitting optic‐fiber catheter through an anchor bolted to the surgical target.^154^ The laser diffuser delivers thermal energy, and MR thermography monitors the temperature of tissues and calculates volume of the damaged tissue within a diameter of ~1 mm. Automated safety points prevent excessive heating and ablation of off‐target tissues.^155^ Multiple ablations can be made over the length of a single trajectory, and multiple trajectories can be used to ablate complex lesions (Figure 3).

Usage of MRgLITT in drug‐resistant focal epilepsy began from 2012.^151^ Although it is not the first‐line alternative, MRgLITT has proved useful for specific cases, such as those requiring access to diseased tissues, those with higher risks (e.g., intracranial bleeding), those whose epileptogenic foci are within or close to critical brain functional areas, and those involving repeated resections and multiple recurrences. It could remove epileptogenic foci (e.g., tubers, cortical malformations, cortical dysplasias, and hypothalamic hamartomas) and to disconnect neural circuits, serving as a novel treatment alternative without the hassles of an open surgery.^148^, ^156^, ^157^, ^158^, ^159^, ^160^, ^161^, ^162^, ^163^

Advantages of MRgLITT over other noninvasive modalities include the ability to monitor an otherwise blind surgical procedure in real time, immediate ablation without a known delayed effect, the option of not using general anesthesia, a shorter postoperative hospital stay, and no need of intensive care unit monitoring typically.^164^ More importantly, MRgLITT also allows access of deep lesions that are otherwise inoperable without damaging overlying eloquent cortex and white matter tracts.^165^ Sparing superficial brain tissues may obviate cognitive deficits subsequent to traditional anterior temporal lobectomy.^166^ In addition, some patients who hesitate to undergo elective epilepsy surgery may have chance with this less invasive surgery or procedure. As a result, MRgLITT has been a hot area of active research for various epileptic lesions, such as hippocampal sclerosis, cortical dysplasia, tuberous sclerosis, periventricular nodular heterotopia, hypothalamic hamartomata, cavernous cerebral malformations, CNS neoplasms, and radiation necrosis.

### 4.3 Safety issues in MRgLITT

Besides a clinically available surgical laser, main components of MRgLITT also include an FDA‐cleared surgical laser ablation system and an MRI‐based image‐processing workstation. In the entire system, the working laser and a cooled laser applicator system are combined with an image‐processing monitor, so that MRgLITT can monitor surgical effects in real time.^160^ The MRI‐compatible laser applicator comprises of a fiber optic applicator, which contains a flexible outer light transmitting cooling sheath and an inner light‐diffusing tip. Along the axis of precise diffusing elements, the laser applicator can produce a roughly cylindrical to ellipsoid distribution pattern in the tissue.^167^, ^168^ During the procedure, targeted tissue is superfused with sterile, room temperature saline through a peristaltic roller pump connected to the applicator. In this way, the laser fiber and adjacent tissues are continuously cooled during the operation and tissue carbonization can be avoided.^160^

During the process laser ablation, serial MR thermal images (MRTIs) are taken to estimate areas of the ablation tissues in near real time.^169^ As proved in previous studies^170^, ^171^, ^172^, it is well‐established that proton resonance frequency shift in an observed image is linearly correlated to the change of temperature.^170^, ^171^, ^172^ Therefore, the temperature can be calculated and displayed as color‐coded “thermal” images in the workstation.^171^ The longitudinal temperature data over time in each voxel are analyzed to estimate the rate of thermal tissue destruction using an Arrhenius equation.^172^ The time‐ and temperature‐dependent rates of protein denaturation are also considered to achieve optimal degree of cellular death. Furthermore, with a pre‐set upper limit for the temperature in each voxel, the laser would automatically shut off once the upper limit is exceeded to avoid undesired tissue damage.^170^

In a recent procedural safety and hospitalization study^173^ after laser ablation of abnormal neurological tissue, 100 patients were followed up for 30 days. Overall, the safety profile in this registry appeared acceptable. A total of 4 adverse events were related to surgical manipulation, such as wound dehiscence, subdural hematoma, bacteremia, and intraventricular hemorrhage. There were 5 adverse events potentially attributable to laser ablation, such as neurological deficits, postoperative seizure, increased peri‐LITT edema, acute intraparenchymal hemorrhage after the procedure, and delayed intraparenchymal hemorrhage. As a matter of fact, nearly half of the treated lesions were considered difficult to access through conventional surgical approaches. These results highlight the importance of a prospective registry for assessing the real‐world uses, outcomes and the clinical potential of an emerging novel technology like LITT, as compared to the more restricted and often less generalizable data associated with randomized clinical trials (or for patient populations not amenable to randomization). Notably, in our own experience, the complication rate of LITT is lower than that with open craniotomies in treating poorly accessible tumors. Average blood loss was also trivial with LITT, consistent with the minimally invasive nature of this technique.

Besides what we have mentioned, there are also other minimally invasive surgeries similar to LITT, such as radiofrequency ablation,^174^, ^175^, ^176^ gamma knife,^177^, ^178^, ^179^ and high intensity focused ultrasound ablation.^180^, ^181^, ^182^, ^183^, ^184^, ^185^ Due to the limitation of the space in the manuscript and their limited applications in epilepsy surgery, we do not expand this information in detail. The summarization and comparison of their respective advantages and disadvantages are listed in Table 1.

**TABLE 1:** Comparison of laser ablation and other minimally invasive / noninvasive ablation techniques

Operation	Radiofrequency ablation	Gamma knife	High intensity focused ultrasound ablation	Laser‐induced interstitial thermotherapy
Working principle	The electrode needle was inserted into the tissue, generating ions in the target tissue that vibrate at high speed in the RF electric field. Heat produced by friction makes the local tissue degenerate and coagulate.	Gamma ray application by geometric focusing and stereotactic method. The planned dose of gamma ray is focused on the target tissue, producing one‐time, fatal destruction.	High intensity ultrasound focused on the target tissue. The thermal effect of the ultrasound leads to instantaneous tissue necrosis and coagulation.	After the laser irradiates the tissue, the light energy is converted into heat energy. Once ablation temperature is reached, the tissue will undergo coagulation and necrosis.
Advantage	It has been widely used and accumulated much practical experience.	Non‐invasive	Not an operation; non‐invasive	Accurate and intuitive by real‐time MRI monitoring; conformal and can adapt to various shapes of tumors by designing the laser output range; suitable for lesions that are deep inside or close to the functional area; the ablation range is large, up to 4 cm; can do both single path with multiple locations and multipath ablation.
Disadvantage	The ablation size was small and cannot be accurately controlled; not conformal; the ablation boundary could not be monitored; invasive, although minimal.	High risk of radiation damage to the tissue around the lesion; associated with serious edema; long procedure duration; treatment is incomplete with high rate of relapse.	Small ablation range; not conformal; high risk of damage in the surrounding tissue; incomplete treatment and high rate of relapse.	Invasive, although minimal.

### 4.4 Clinical use of MRgLITT in drug resistance focal epilepsy

Emerging data support the safety and clinical efficacy of LITT as treatment for a spectrum of neurosurgical pathologies including low‐ and high‐grade gliomas, brain metastases, radiation necrosis, and seizure foci. However, these datasets are mostly small (<50 patients) and/or retrospective reports of single‐institutional series. Moreover, there is significant heterogeneity in these studies in terms of quality assurance, definition of complications, and data validation. These challenges limit the generalizability of the reported data. Additionally, interpretation of this dataset is often confounded by various forms of biases inherent in retrospective, institutional studies.

For example, in patients with drug‐resistant mesial temporal lobe epilepsy (mTLE), MRgLITT might provoke the decline of memory in adult patients. mTLE per se is associated with altered mitochondrial respiratory chain complex enzyme activities, which may explain the susceptibility to cognitive impairment. Nevertheless, in appropriately selected case whose epileptogenic zone is clearly identified by well‐localized intracranial EEG, MRgLITT as an initial procedure adjunctive to open surgery after MRgLITT could be beneficial.^186^ Although open temporal lobe surgery for mTLE proves to be a well‐tolerated procedure that improves quality of life,^152^ it can induce unrecognized neurocognitive deficits. According to previous reports, the deficits are usually caused by the collateral damage in the temporal lobe, and they usually occur when mesial temporal structures are approached. With the application of MRgLITT, a less invasive stereotactic procedure, this damage may be avoided.^187^ Thus far, some believe that MRgLITT promulgates as an alternative approach alone for the obliteration of seizure foci. It may play a better adjunctive role than other procedures in combination with open surgery. MRgLITT also occasioned opportunities of conducting comprehensive pathological analyses for dysplastic brain tissue before the open resect surgery.^156^, ^164^, ^188^, ^189^, ^190^, ^191^

In other studies,^192^, ^193^ long‐term outcome MRgLITT alone was evaluated from follow‐up (10 months, ranging 1–39 months) study in pediatric patients showed that more than 78% of patients were seizure free or showed improvement in a mixed cohort of patients with tuberous sclerosis, periventricular heterotopias, focal cortical dysplasias, hypothalamic hamartomas, and mesial temporal sclerosis. Notably, the degree of quadrantanopsia (common in pediatric seizure patients after open surgery) is limited and has little serious influences on patients’ life qualities.^194^, ^195^ Major complication with regards to MRgLITT is the development of new neurological defects. The most prevalent is motor deficits, including facial droop, gait instability paresis, and hemiplegia. Other defects include accidental cognitive decline or vision problems like diplopia within 6 months.^163^ Hemorrhagic complications are relatively severe but rare (incidence less than 1%), almost always related to impingement on vascular structures during stereotactic placement of a laser probe. Since there are few patients in these cohorts, we could not conclude any statistically significant conclusions with the limited number of patients.

### 4.5 A case report on the application of MRgLITT for insular epileptic seizure (workflow case)

As an addition of the usage of MRgLITT in seizure, we here report an insular epileptic seizure case successfully treated by MRgLITT.

**FIGURE 4:**
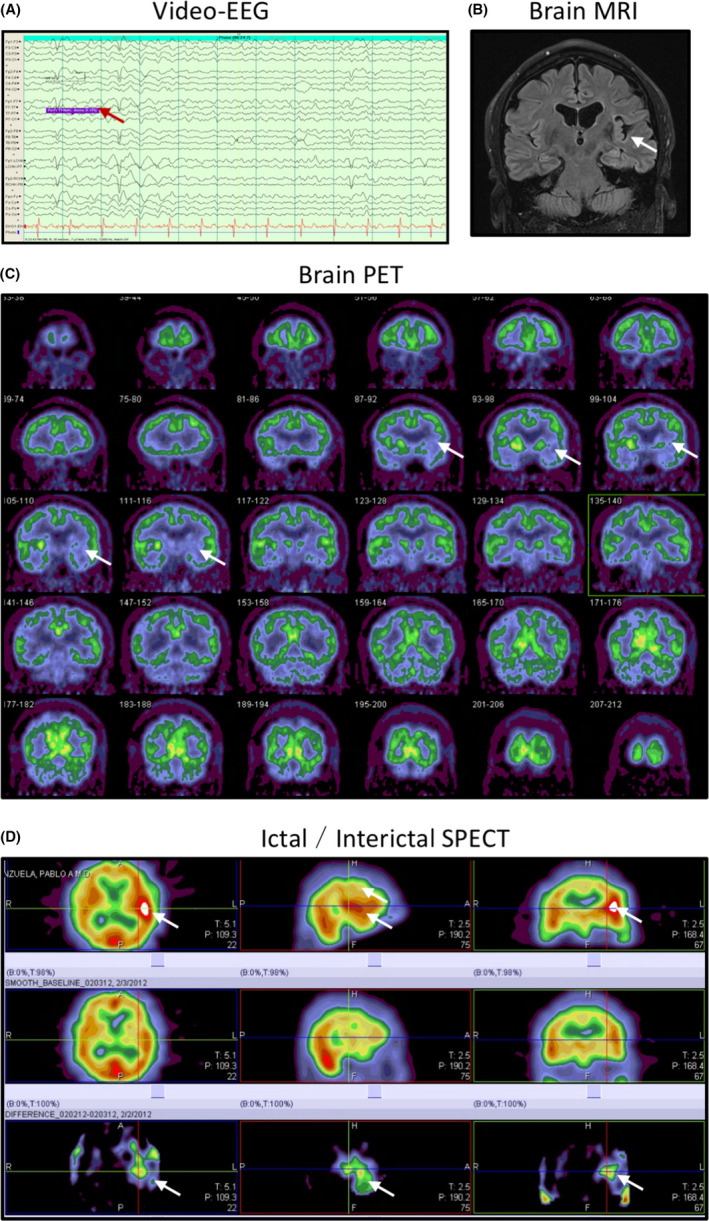
Pre‐operative evaluation of the patient for localizing the epileptogenic focus. (A) A typical seizure event on video‐EEG with left hemispheric diffuse onset and evolution. (B) Encephalomalacia in the left insula revealed by brain MRI. (C) Hypometabolism in the left insula revealed by PET scan of the brain. (D) Hyperperfusion in the region of MRI‐defined encephalomalacia in left insula after an ictal SPECT injection during a typical complex partial seizure

**FIGURE 5:**
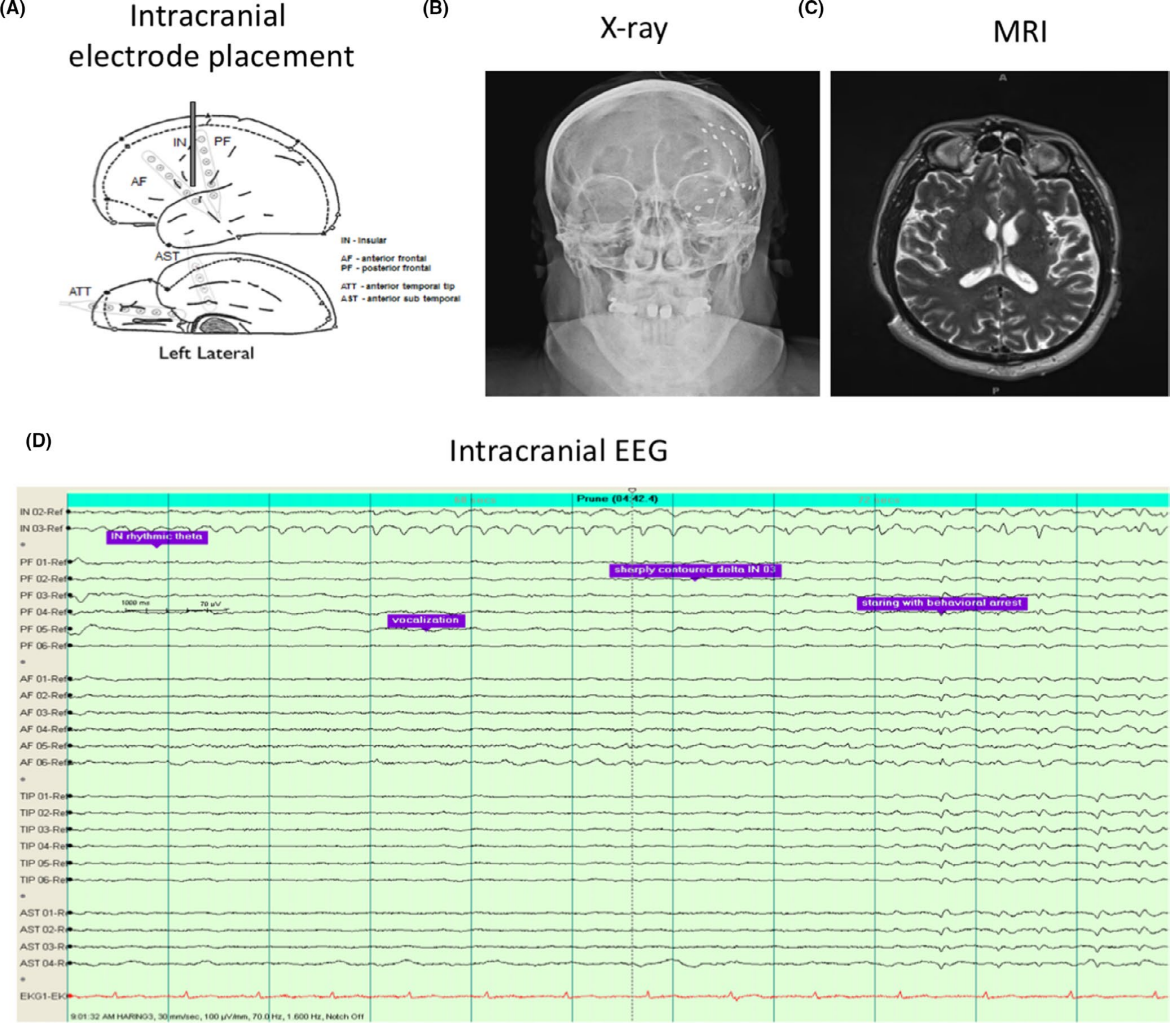
Intracranial electrode placement and EEG recordings. Stereotactic placement of left insular depth electrodes and left front‐temporal strip electrodes shown by (A) pattern diagram, (B) X‐ray, and (C) MRI. (D) The intracranial video‐EEG showed seizure events with left insular onset. Ictal discharges were marked by purple color bars

**B.G.** A 54‐year‐old man presented with a 17‐year history of refractory complex partial seizures. The main clinical symptoms of the patient included a metallic taste aura and behavioral arrest, stereotypical automatism with the left hand covering the face, deep breathing with ongoing automatism, and unresponsiveness with decreased awareness. This was followed by secondary generalized tonic‐clonic seizure with loss of consciousness, vocalization, head deviation, body stiffening, and jerking. Postictal cough was present. This patient had failure of five antiepileptic drugs. After undergoing video‐EEG, the above events were shown as diffuse onset and subsequent evolution in left hemisphere (Figure 4A). Brain MRI suggested that encephalomalacia in left insula (Figure 4B). A PET scan revealed hypometabolism in the left insula (Figure 4C). Ictal SPECT of a seizure episode showed hyperperfusion within the MRI‐defined encephalomalacia in left insula (Figure 4D). Left insular depth electrodes and front‐temporal strip electrodes were placed with stereotactic MRI navigation (Figure 5A–C). Intracranial EEG revealed 5 episodes of complex partial seizures with onset in the left insular and propagation to the left temporal and frontal regions (Figure 5D).

**FIGURE 6:**
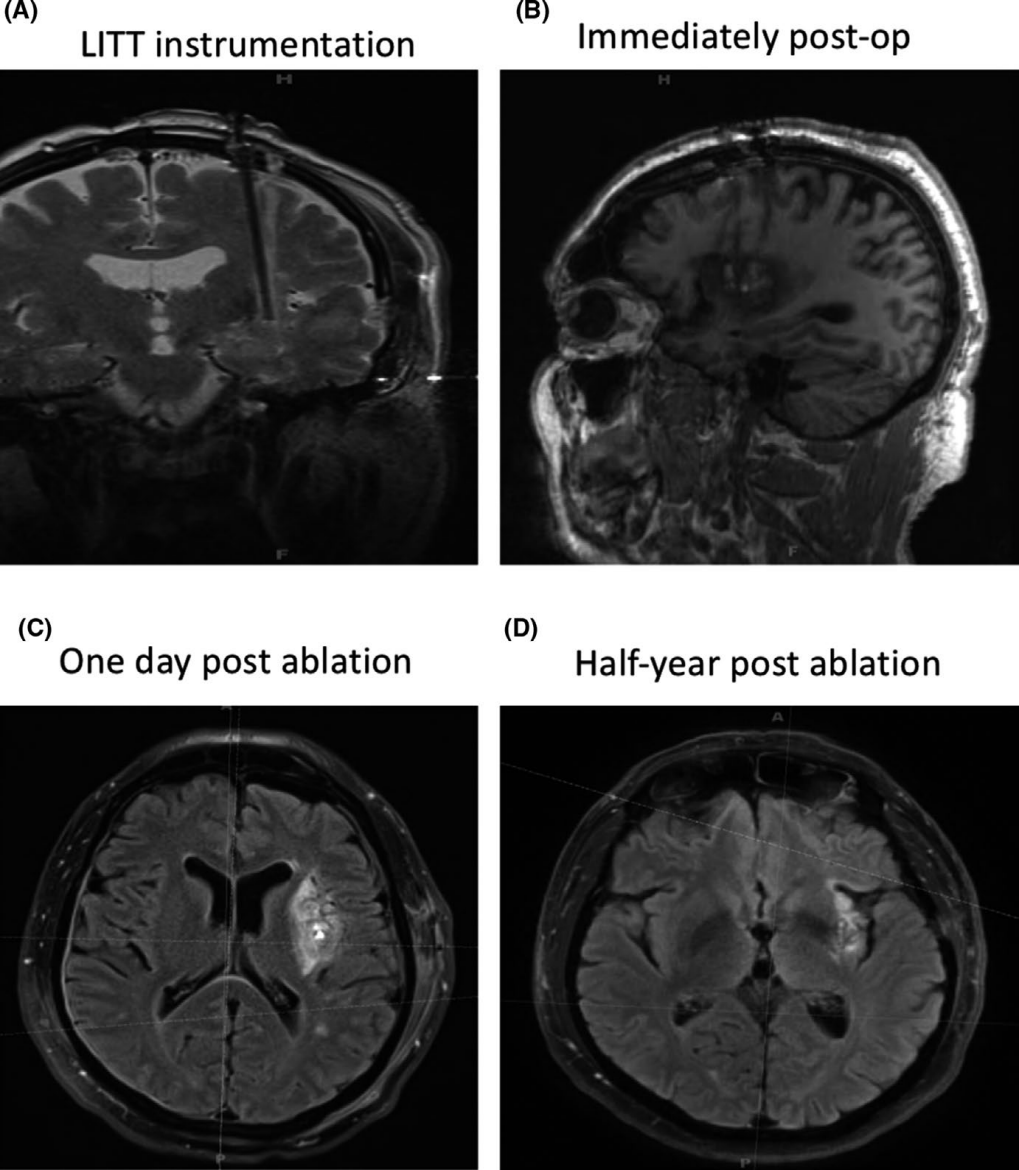
Brain MRI follow‐up of the patient after ablation. (A) LITT instrumentation shown by coronal image of brain MRI. (B) Brain MRI manifestation immediately postablation on sagittal image. Brain MRI manifestations one day (C) and half‐year (D) postablation on axial image

LITT was performed to ablate the left anterior and posterior insular lesion, targeting the epileptogenic area as well as surrounding encephalomalacia. Symptoms of anxiety and mild dysarthria were reported after the operation, which gradually recovered during the 6‐month follow‐up. No significant hemorrhage or unexpected brain damage was observed on MRI during the 6‐month follow‐up (Figure 6). He remained seizure‐free two years after the operation.

In summary, this is a case of insular seizure with relatively diffuse onset on scalp EEG, ictal SPECT for localization, and ultimately LITT for ablation of the epileptogenic lesion, suggesting the clinical value of such diagnostic and treatment approaches in insular seizures. MRgLITT ablation is an exciting, novel, and minimally invasive technique for the treatment of epilepsy when the epileptogenic lesion can be clearly localized. Long‐term outcomes should be further followed up and evaluated to validate the efficacy and safety of this technique.

## 5 CONCLUSION

With the development of our understanding on epilepsy and novel techniques, minimally invasive surgery has increasingly become a surgical alternative for patients with refractory focal epilepsy lesions. MRgLITT is one of them. In recent decades, the laser technique for the care of neurosurgical patients has significantly improved, allowing MRgLITT an efficient and effective alternative for ablation of the epileptogenic zone, as well as for disconnection procedures in patients with intractable epilepsy. It could significantly improve seizure control. As a minimally invasive alternative, MRgLITT exhibits equal efficacy, which may encourage the epileptologists and patients to consider this procedure at earlier stage of the disease when they otherwise would hesitate on all invasive procedures.

Current reports showed that MRgLITT is associated with relatively fewer complications, such as temporary neurological defects. However, the lack of large prospective studies makes it hard to conclude for now. Other problems include the lack of a consensus on the dose of thermal energy per unit volume for target tissue ablation, although the use of thermal energy based on MRI thermography‐visual feedback is sufficient to assure safety. In addition, there is also a lack of standard surgical protocols or workflows.

Collectively, we believe MRgLITT has prosperous future as a single treatment, or in combination with traditional open surgery. Prospective trials on its safety and a standard protocol are needed in future research.

## CONFLICT OF INTEREST

No potential conflict of interest was reported by the authors.

## AUTHOR CONTRIBUTIONS

WS and XM participated in literature search, figures, study design, data collection, data analysis, data interpretation, writing, critical approval of the final report, and funding. QW had full access to the data and take responsibility for the integrity of the data and the accuracy of analysis. EH participated in data collection, writing and critical approval of final report. We confirm that the manuscript has been read and approved by all named authors and that there are no other persons who satisfied the criteria for authorship but are not listed. We further confirm that all of us have approved the order of authors listed in the manuscript.

## Data Availability

The data that support the findings of this study are available on request from the corresponding author. The data are not publicly available due to privacy or ethical restrictions.
